# Transmission Disrupted: Modeling Auditory Synaptopathy in Zebrafish

**DOI:** 10.3389/fcell.2018.00114

**Published:** 2018-09-11

**Authors:** Katie S. Kindt, Lavinia Sheets

**Affiliations:** ^1^Section on Sensory Cell Development and Function, NIDCD/National Institutes of Health, Bethesda, MD, United States; ^2^Department of Otolaryngology, Washington University School of Medicine, St. Louis, MO, United States

**Keywords:** zebrafish model system, hair cells (HCs), ribbon synapse, deafness/hearing loss, synaptic transmission

## Abstract

Sensorineural hearing loss is the most common form of hearing loss in humans, and results from either dysfunction in hair cells, the sensory receptors of sound, or the neurons that innervate hair cells. A specific type of sensorineural hearing loss, referred to as auditory synaptopathy, occurs when hair cells are able to detect sound but fail to transmit sound stimuli at the hair-cell synapse. Auditory synaptopathy can originate from genetic alterations that specifically disrupt hair-cell synapse function. Additionally, environmental factors such as noise exposure can leave hair cells intact but result in loss of hair-cell synapses, and represent an acquired form of auditory synaptopathy. The zebrafish model has emerged as a valuable system for studies of hair-cell function, and specifically hair-cell synaptopathy. In this review, we describe the experimental tools that have been developed to study hair-cell synapses in zebrafish. We discuss how zebrafish genetics has helped identify and define the roles of hair-cell synaptic proteins crucial for hearing in humans, and highlight how studies in zebrafish have contributed to our understanding of hair-cell synapse formation and function. In addition, we also discuss work that has used noise exposure or pharmacological mimic of noise-induced excitotoxicity in zebrafish to define cellular mechanisms underlying noise-induced hair-cell damage and synapse loss. Lastly, we highlight how future studies in zebrafish could enhance our understanding of the pathological processes underlying synapse loss in both genetic and acquired auditory synaptopathy. This knowledge is critical in order to develop therapies that protect or repair auditory synaptic contacts.

## Introduction

**FIGURE 1:**
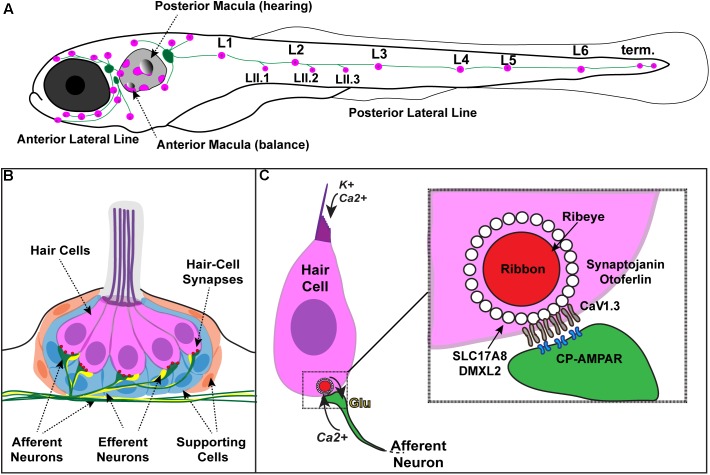
Zebrafish hair cells and ribbons synapses. **(A)** Schematic depicts a larval zebrafish. Pink patches outline the location of hair cells in the inner ear required for hearing and balance, as well as hair cells in the lateral-line system. Green patches represent the location of the anterior and posterior lateral-line ganglia. The cell bodies of neurons in these ganglia project to and innervate hair cells in the lateral line. **(B)** An overview of the anatomy of a single patch of hair cells in the lateral line, referred to as a neuromast. Hair cells (pink) are surrounded by supporting cells (internal, blue and peripheral, orange) and innervated by both afferent (green) and efferent neurons (yellow). Mechanosensory hair bundles (purple) at the apex of hair cells project out into the water to detect local water flow. **(C)** Diagram of a single hair cell. Hair cells are activated when hair bundles are deflected, for example by local water flow. This apical deflection opens mechanosensitive channels allowing in cations including potassium and calcium. This apical activity depolarizes the hair cell, resulting in presynaptic calcium influx and release of glutamate onto the afferent neuron. Inset: magnified view of a hair-cell ribbon synapse. Shown are key evolutionarily conserved synaptic proteins discussed in this review. In hair cells, a presynaptic density called a ribbon (red) helps to recruit synaptic vesicles (white circles) to the synapse near clusters of calcium channels (Ca_V_1.3). The ribbon is made up primarily of the protein Ribeye. Slc17A8 (Vglut3) and DMXL2 (Rbc3a) colocalize in or near synaptic vesicles. Synaptojanin and Otoferlin are also critical for ribbon-synapse function although their precise localization has not been definitively shown.

Sensory hair cells in our inner ear must both reliably transduce and transmit auditory and vestibular stimuli (Harris et al., 1970; Eatock and Fay, 2006; **Figures 1A,B**). Hair cells transduce stimuli when apically localized mechanosensitive channels are activated, leading to graded depolarization of the hair-cell membrane (Gillespie and Walker, 2001; **Figure 1C**). Hair-cells transmit stimuli at the hair-cell synapse. At the synapse, hair-cell depolarization opens voltage-gated calcium channels; calcium influx through these channels drives synaptic vesicle fusion and glutamate release onto innervating afferent nerves (Fuchs, 2005; Moser et al., 2006; **Figure 1C**). If either the hair cells or downstream afferent nerves are damaged or dysfunctional, the pathological consequence is sensorineural hearing loss. Sensorineural hearing loss can be caused by genetic factors, infections, toxins, age and excessive noise, and is the most common form of hearing loss in humans (90% of cases) (Eggermont, 2017; World Health Organization, 2018).

One form of sensorineural hearing loss—auditory synaptopathy—results when hair cell transduction is intact, yet synaptic transmission of sound stimuli from hair cells to downstream afferent nerves is disrupted. Auditory synaptopathy can result from genetic alterations that disrupt molecules requires for synapse function. Additionally, auditory synaptopathy and associated hearing loss can also be acquired though noise exposure (Bharadwaj et al., 2014). Both genetic and acquired auditory synaptopathy stem from dysfunction of specialized synapses in hair cells called ribbon synapses. Ribbon synapses have a unique presynaptic specialization called a synaptic ribbon or ribbon body which tethers synaptic vesicles near calcium channels at the presynaptic active zone (**Figure 1C**). Ribbon synapses are required for transmitting stimuli in a fast and sustained manner needed for precise sensory encoding, and are structurally and functionally unique from classic neuronal synapses (Fuchs, 2005; Moser et al., 2006). Understanding the unique properties of hair-cells synapses is an active area of study, and continued research is necessary in order to define the pathologies underlying auditory synaptopathy.

Our current understanding of how hair-cell synapses function, and the underlying causes of auditory synaptopathy, has been built by genetic studies in several model systems. In particular, the zebrafish model has been used to help identify and define the functions of genes important for hair-cell synapse function. Zebrafish hair cells are remarkably similar to mammalian hair cells at the molecular and cellular level (Coffin et al., 2004). Genetic studies have demonstrated that numerous genes required for hearing and balance in zebrafish are also required in mice and humans (Coffin et al., 2004; Nicolson, 2005; Varshney et al., 2016). In addition to genetic conservation, one significant advantage of studying hair cells and their synapses in zebrafish is the ability to study hair cells *in vivo*. In mammals, the inner ear is encased in bone, making it impracticable to study these sensory cells in their native environment. In contrast, larval zebrafish are transparent, and hair cells are optically accessible in whole larvae. In zebrafish larvae, hair cells are present both within the inner ear and the lateral-line system—a sensory organ used to detect the movement of water. The lateral line is made up of clusters of hair cells called neuromasts that are arranged in series along the fish body and head (**Figures 1A,B**). Neuromast hair cells are particularly advantageous for hair-cell assessment because these cells are located superficially just beneath the fish skin, with their apical hair bundles protruding into the aqueous environment. This access makes it relatively straightforward to apply pharmacological agents, to stimulate the hair cells with fluid-flow, and assess hair-cell structure and function *in vivo*. Moreover, relative to mammals where hair cells mature over several weeks (Kraus and Aulbach-Kraus, 1981; Romand and Varela-Nieto, 2003), the hair cells in zebrafish mature rapidly (<24 h; Kindt et al., 2012; Dow et al., 2015), making it possible to study the entirety of hair-cell development in a single imaging session.

In addition to hair-cell accessibility and rapid development, the zebrafish model is valuable for hearing and balance research because, similar to mice, it is genetically tractable model system. Zebrafish are amenable to rapid genetic modification, including transgenic modification to express tissue specific transgenes encoding fluorescent markers or gene products (Kwan et al., 2007). The use of fluorescent markers is especially useful in the transparent larvae where hair-cell structures can easily be visualized *in vivo* and dynamic cellular processes can be imaged in a live, intact preparation.

In this review, we provide an overview of tools and techniques developed in the zebrafish model to examine hair-cell synapse structure and function. We also describe genetic studies in zebrafish that have helped define the roles of key hair-cell synaptic proteins. Given the recent advances in gene-editing technology, we highlight how zebrafish genetics could be applied to further our understanding of the genetic causes of auditory synaptopathy. Lastly, we outline preliminary studies that have explored the potential for using zebrafish to model noise-exposure and its associated excitotoxicity. We conclude with a discussion on how noise exposure studies in zebrafish could be expanded to further our understanding of the specific pathological changes that lead to acquired, noise-induced auditory synaptopathy.

## Toolkit to Assess Hair-Cell Synapse Function and Morphology in Zebrafish

Over the years, experimental techniques have been developed to study hair cells and hair-cell synapses in zebrafish. These techniques include: optical and ultrastructural analyses to visualize hair-cell synapse morphology, and functional assays to examine how hair cells transduce and transmit sensory stimuli. In the section below, we outline these methods and tools.

### Morphological Analysis of Hair-Cell Synapses in Zebrafish

Genetic mutations or environmental insults such as noise exposure can specifically affect the spatial organization of hair-cell synaptic structures (Paquette et al., 2016; Ryan et al., 2016; Song et al., 2016). In the mammalian inner ear, hair-cell synapses are commonly characterized ultrastructurally using transmission electron microscopy (TEM) to examine synapses in either single or serial-sections. In addition, these synapses can be examined using confocal microscopy to visualize immunolabel of hair-cell synaptic proteins (Liberman et al., 2011; Valero et al., 2017; Becker et al., 2018; Jean et al., 2018).

**FIGURE 2:**
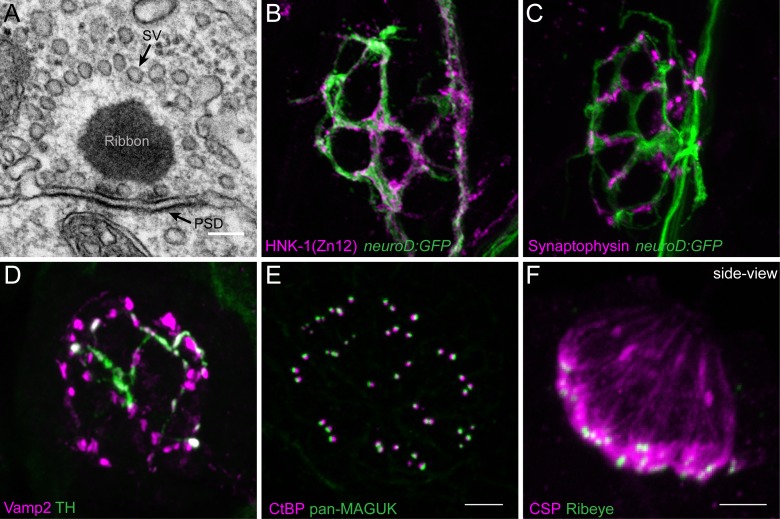
Morphological examination of hair-cell synapses in zebrafish. **(A)** Classically, transmission electron microscopy (TEM) has been used to visualize hair-cell synapses. Shown is a micrograph of a hair-cell synapse from a zebrafish lateral-line hair cell. In this micrograph, the presynaptic ribbon is a dark spherical density. Surrounding the presynaptic ribbon are synaptic vesicles (SV). Beneath the presynaptic ribbon along the plasma membrane is the postsynaptic density (PSD). **(B)** The *neurod:GFP* transgene (green) can be used to label the afferent neurons innervating lateral line (shown in **A,B**), as well as afferents that innervate inner-ear hair cells. Afferent fibers can be labeled with the commercial antibody HNK-1/Zn12 (pink). **(C)** *Neurod:GFP* (green) can be co-labeled with a Synaptophysin antibody (pink) to label both afferent fibers and all efferent synapses respectively. **(D)** Efferent synapses, which can also be labeled with a Vamp2 antibody (pink), can be further sub-classified by a co-label such a tyrosine hydroxylase (TH, green; white overlap indicates dopaminergic synapses). **(E)** Pre- and post-synaptic densities can be labeled with CtBP (pink) and pan-MAGUK (green) antibodies respectively. **(F)** Synaptic vesicles, labeled with cysteine string protein (CSP, pink) are enriched at the basolateral membrane of hair cells near synaptic ribbons labeled with Ribeye antibody (green). Scale bar = 100 nm in **(A)** and 5 μm in **(E)** (for **B–E**) and **(F)**.

Similar to work in mammals, precise ultrastructural measurements can be obtained from zebrafish hair-cell synapses using TEM (**Figure 2A**). For example, in zebrafish, the synaptic ribbon can be seen clearly in TEM as an electron-dense region that is adjacent to the postsynaptic density on the innervating afferent neuron (**Figure 2A**, ribbon and PSD). TEM is the most accurate way to determine the size of the synaptic ribbon. TEM can also be used to visualize the synaptic vesicles tethered to the synaptic ribbon and near the active zone (**Figure 2A**, SVs). Currently TEM is the only method able to quantify the number and distribution of these synaptic vesicles populations. While these ultrastructural measurements are valuable, preparing, sectioning, imaging and analyzing TEM samples requires considerable time and effort. Moreover, in most cases, TEM is only able to capture a subset of synapses within each hair-cell organ.

In contrast to electron micrographs, quantitative analysis of immunolabeled epithelial whole mounts provide the advantage of being able to examine synaptic features of large numbers of hair cells. This advantage has been used by a number of groups to characterize relative variances in size and morphologies of pre- and postsynaptic components in mammalian and zebrafish hair-cell organs (Wong et al., 2014; Paquette et al., 2016; Suli et al., 2016; Becker et al., 2018; Jean et al., 2018). Further benefits of using the larval zebrafish lateral-line system for quantitative imaging of immunolabeled structures are twofold. First, the relative simplicity; each neuromast contains 10–16 hair cells with ∼3 synapses per cell of similar morphology, making comparative analyses of synapses in numerous lateral-line organs straightforward (Sheets et al., 2012; Suli et al., 2016). This in contrast to mammalian auditory hair cells where the number and size of synapses can vary among individual hair cells depending on location within the Organ of Corti—the sensory organ for hearing (Meyer et al., 2009). Second, the synaptic ribbons in the zebrafish lateral line (TEM, circular 300 nm diameter; Suli et al., 2016) are on average larger compared to those in mammalian auditory hair cells (mouse TEM length (longest axis of the ribbon) ∼120 nm prehearing and ∼170 nm hearing; Wong et al., 2014)). These larger synaptic ribbons are near the resolution of light microscopy (∼200–270 nm) making it relatively straightforward to resolve these structures.

A previous challenge using the zebrafish lateral-line system for analysis of immunolabeled synaptic structures was a scarcity of antibodies that interact with zebrafish hair-cell synaptic proteins. In recent years a number of commercially available antibodies have been identified that label zebrafish synaptic components including synaptic vesicles (**Figure 2F**, CSP), synaptic ribbons (**Figure 2E**, CtBP), postsynaptic densities (**Figure 2E**, MAGUK), afferent (**Figure 2B**, ZN-12) and efferent (**Figure 2C**, Synaptophysin and **Figure 2D**, Vamp2 and TH) lateral-line neurons, and glutamate receptor subunits (Summary of Abs: **Table 1**). Immunolabels can be used in combination with transgenic lines that specifically mark cell types of interest such as hair cells, glia-like supporting cells, as well as the innervating neurons (**Figures 2B,C**, *neurod:GFP*; Obholzer et al., 2008; Behra et al., 2012, p. 1; Tabor et al., 2014; Toro et al., 2015).

**Table 1:** Commercially available antibodies labeling zebrafish hair-cell synaptic proteins.

Antigen	Structure Labeled	Host	Antibody type	Dilution	Company/Catalog #	Reference
Acetylated Tubulin	Afferents and hair cells	Mouse	Monoclonal IgG2b	1:5000	Sigma/T7451	Harris et al., 2003; Murakami et al., 2003; Obholzer et al., 2008; Kindt et al., 2012
Calretinin	Afferent processes	Mouse	Monoclonal IgG1	1:1000	Swant/6B3	Pei et al., 2016
Choline Acetyltransferase	Cholinergic efferent terminals	Goat	Polyclonal	1:500	Millipore Sigma/AB144P	Zhang et al., 2018
CtBP (1&2)	Synaptic ribbons	Mouse	Monoclonal IgG2a	1:1000	Santa Cruz/sc-55502	Lv et al., 2016
Cysteine String Protein	Synaptic vesicles	Rabbit	Polyclonal	1:1000	Millipore Sigma/AB1576	Lin et al., 2016
Gria 4	AMPA glutamate receptor subunit	Rabbit	Polyclonal	1:400	Millipore Sigma/AB1508	Sheets, 2017
Grik 2	Kainate glutamate receptor subunit	Rabbit	Polyclonal	1:400	Fitzgerald Industries/70R-1522	Sheets, 2017
Grik 4	Kainate glutamate receptor subunit	Rabbit	Polyclonal	1:400	Genway Biotech Inc./GWB-DA6FF7	Sheets, 2017
Grin 1	NMDA glutamate receptor subunit	Mouse	Monoclonal IgG2b	1:1000	Synaptic Systems/114011	Sheets, 2017
Human Natural Killer-1	Afferent processes	Mouse	Monoclonal IgG1	1:500	Developmental Studies Hybridoma Bank/ Zn-12	Mo and Nicolson, 2011
MAGUK	Postsynaptic densities	Mouse	Monoclonal IgG1	1:500	NeuroMab (UC Davis)/ 75-029	Sheets et al., 2011; Lv et al., 2016
NSF	Hair cells and afferent process	Rabbit	Monoclonal	1:50	Cell Signaling/3924	Mo and Nicolson, 2011
Otoferlin	Hair cells	Mouse	Monoclonal IgG2a	1:500	Developmental Studies Hybridoma Bank/ HCS-1	Faucherre et al., 2009; Goodyear et al., 2010
Rab3	Hair cells and efferent neurons	Mouse	Monoclonal IgG1	1:1000	Synaptic Systems/107011	Einhorn et al., 2012
Synaptophysin 1	Efferent terminals	Mouse	Monoclonal IgG1	1:1000	Synaptic Systems/ 101 011	Toro et al., 2015
Tyrosine Hydroxylase	Dopaminergic efferents	Mouse	Monoclonal IgG2a	1:1000	Vector labs/ VP-T489	Toro et al., 2015
Vamp 2	Efferent terminals	Rabbit	Polyclonal	1:500	Genetex/ GTX132130	Zhang et al., 2018


**FIGURE 3:**
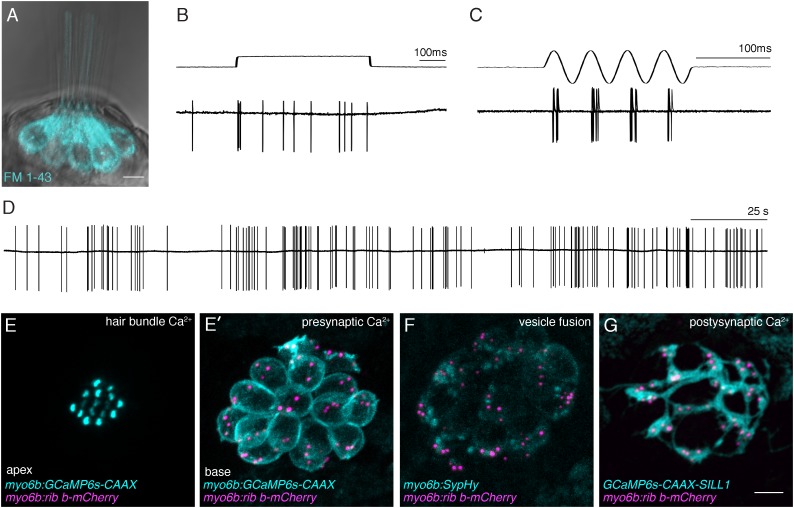
Functional analysis of hair-cell synapses in the zebrafish lateral line. **(A)** In the lateral line, rapid uptake of the vital dye FM 1-43 (cyan), shown in this example, indicates mechanotransduction is intact in these hair cells. **(B–D)** Extracellular recordings from the afferent cell bodies in the posterior lateral-line ganglia can be used as a read-out of synapse function. During these afferent recordings, evoked spikes can be detected when innervated hair cells are stimulated along their axis of sensitivity **(B,C)**. Step stimuli (**B**, anterior step shown) are useful to quantify spike number and the timing of the first spike. Sine stimuli (**C**, anterior-posterior sine stimulus shown) are useful to quantify the precision of spike timing within the waveform. Note that each afferent only responds to one direction of stimuli (for example the anterior but not posterior phase of the sine wave in **C**). Even in the absence of stimuli there is spontaneous spiking in that can be used as a read-out of synaptic function **(D)**. **(E,E’)** A transgenic line expressing a membrane-localized calcium indicator GCaMP6s (cyan) can be used to detect mechanotransduction-dependent calcium influx in apical hair bundles **(E)** and calcium influx at synaptic ribbons **(E’)** when apical and basal planes are imaged respectively. **(F)** Transgenic fish expressing SypHy, an indicator of vesicle fusion can be used to detect presynaptic vesicle fusion at hair-cell synapses. **(G)** GCaMP6s can also be used to detect postsynaptic calcium activities in the afferent process beneath hair cells. All of the transgenic approaches outlined in **(E–G)** can be used in combination with a transgenic line that marks synaptic ribbons via a Ribeye b-mCherry fusion protein. The scale bar in **(A,G)** = 5 μm.

Transmission electron microscopy and immunostaining provide important information regarding synaptic structure and the localization of proteins at hair-cell synapses. Unfortunately, there are not antibodies for all synaptic proteins, and these approaches do not provide temporal information regarding the dynamics of these proteins within hair cells. Therefore, transgenic fish lines expressing fluorescently tagged synaptic proteins provide a powerful way to determine the localization of these molecules *in vivo* (Trapani et al., 2009; Sheets et al., 2011; Einhorn et al., 2012). For example, transgenic fish expressing fluorescently tagged Ribeye, one of the main structural components of synaptic ribbons, have been used to identify the location of ribbons in developmental analyses and functional imaging experiments (*Tg[myo6b:ribeye-mcherry];* **Figures 3F,G**; Pujol-Martí et al., 2014; Zhang et al., 2018). More recent work has used tagged proteins to investigate the structural dynamics of synaptic ribbons. For example, fish expressing fluorescently tagged Ribeye have been used along with fluorescence recovery after photobleaching (FRAP) to determine the stability and turnover of Ribeye within synaptic ribbons, and the exchange of Ribeye between synaptic ribbons (Graydon et al., 2017; Chen et al., 2018). In the future, the creation of additional zebrafish transgenic lines will provide a valuable resource in this *in vivo* model to study the localization and dynamics of hair-cell synaptic proteins.

### Assays for Hair-Cell Mechanotransduction in Zebrafish

Hair-cell function occurs primarily within two main structural domain, the apical hair bundle and the basal ribbon synapse. These structural domains are required for mechanotransduction and neurotransmission respectively. In response to sensory stimuli, apical hair bundles are deflected; this deflection opens mechanosensitive ion channels. This apical activity is essential to initiate hair-cell depolarization, and opening of calcium channels at the synapse, leading to sensory-evoked neurotransmission. Therefore, these two domains are functionally linked and, in order for proper hair-cell synapse function, apical hair bundles must be intact and functional. To assay for normal mechanotransduction in zebrafish, microphonic potential measurements and FM 1-43 dye labeling can be used (**Figure 3A**; Nicolson et al., 1998; Seiler et al., 2004). While these measurements are most straightforward in lateral-line hair cells, they have also been adapted to examine hair cells in the zebrafish inner ear (Tanimoto et al., 2011). In the lateral line, microphonic potentials are recorded by placing an extracellular electrode near the apical hair bundles of an individual neuromast. Hair bundles are then deflected with a fluid jet and the flow of current into mechanosensitive ion channels in the bundles of all hair cells within a neuromast can be measured (Trapani and Nicolson, 2010; Olt et al., 2016b). For FM 1-43 analysis, larvae are briefly immersed in the vital dye FM 1-43. This dye rapidly enters hair cells when mechanosensitive ion channels are functional (**Figure 3A**; Seiler and Nicolson, 1999). These combined methods provide a way to ensure that hair-cell mechanotransduction is intact, and represent important assays in the characterization of zebrafish auditory and vestibular mutants. For example, mutants without microphonic potentials or FM 1-43 label can be classified as mechanotransduction mutants. In contrast, mutants with intact microphonic potentials and FM 1-43 label indicate that there is a disruption downstream of mechanotransduction. Importantly, a subset of zebrafish mutants with normal mechanotransduction have been shown to be required for hair-cell synapse function. (See the genetics section below; Nicolson et al., 1998; Sidi et al., 2004; Obholzer et al., 2008; Trapani et al., 2009; Einhorn et al., 2012). Overall, these assays are useful in determining whether specific mutations disrupt components of the transduction apparatus, or potentially affect a process downstream, including hair-cell transmission.

### Electrophysiological Approaches to Study Hair-Cell Synapses in Zebrafish

In addition to microphonics and FM 1-43, several additional methods have been established to assay hair-cell function, and specifically hair-cell synapse function in zebrafish. These methods utilize either electrophysiology or imaging-based approaches (Olt et al., 2016b; Zhang et al., 2016). In general, imaging approaches offer superior spatial resolution, while electrophysiological recordings offer greater sensitivity. Both of these methods have proved invaluable in the analysis of molecules required for hair-cell synapse function in zebrafish.

To study presynaptic function, the gold standard in the hair-cell field is whole-cell patch-clamp recordings. Using this approach, stimulatory voltage steps can be applied to electrically isolated individual hair cells in order to obtain important information on synapse function; for example, calcium currents reliant on presynaptic calcium channels can be isolated and characterized (Brandt et al., 2005; Ricci et al., 2013; Olt et al., 2014). In addition, increases in cell-membrane capacitance that are associated with vesicle fusion can also be measured. These hair-cell recording have been pioneered in zebrafish by several groups (Ricci et al., 2013; Olt et al., 2014). Using this method, both presynaptic calcium currents and capacitance changes have been recorded in larval and juvenile zebrafish lateral-line hair cells (Olt et al., 2014, 2016a).

In addition to hair-cell electrophysiological measurements, recordings from the afferent posterior lateral-line ganglia have also been used to characterize hair-cell synapse function (Trapani and Nicolson, 2010). Within each neuromast there are two populations of hair cells that respond to water flow in two directions along a single axis. Afferent neurons that innervate neuromasts form bouton-like synapses on multiple hair cells that respond to one of these two directions (Nagiel et al., 2008; Faucherre et al., 2009; Sheets et al., 2011; Pujol-Martí et al., 2014). Therefore, electrophysiological recording from afferents represent the summed output of several hair cells within a neuromast.

Afferent recordings in zebrafish have primarily measured extracellular currents from afferent cell bodies in a loose-patch configuration. Initially, a successful afferent recording is identified by detection of spontaneous spiking that results from hair-cell neurotransmission in the absence of stimuli (**Figure 3D**; Trapani and Nicolson, 2011). Spontaneous spike rate on its own has been shown to be an important feature of synaptic-release properties in both zebrafish and mammals (Pfeiffer and Kiang, 1965; Merchan-Perez and Liberman, 1996; Furman et al., 2013; Sheets et al., 2017). After identifying a spiking afferent neuron, a fluid-jet can be used to deflect the hair bundles of each neuromast along its axis of sensitivity in order to identify an afferent neuron and neuromast that are synaptically paired (**Figures 3B,C**). Each afferent will respond to a single direction along the neuromast’s axis of sensitivity (**Figures 3B,C**). Once this pairing is identified, many features relevant to hair-cell synapse function can be quantified using these recordings. For example, during fluid-jet stimulation, the timing and number of afferent spikes can be recorded. While spike number can provide important information on vesicle release and replenishment (Trapani et al., 2009; Einhorn et al., 2012; Sheets et al., 2017), measurements such as first spike latency (time from stimulus to the first spike) can provide important information on the timing of vesicle release (Sheets et al., 2017). Additionally, during a sinusoidal stimulus where the fluid-jet is used to stimulate both populations of hair cells within a neuromast along its axis of sensitivity, phase-locking can be measured (**Figure 3C**). Phase-locking assesses how consistently the afferent neuron spikes during a particular phase of the sine stimulus and reflects the fidelity of neurotransmission. Overall, afferent recordings have been able to resolve subtle yet important differences in zebrafish mutants that alter hair-cell synapse function (Obholzer et al., 2008; Trapani et al., 2009; Einhorn et al., 2012).

### Using Functional Imaging to Study Hair-Cell Synapses in Zebrafish

Because the zebrafish model system is genetically tractable, work in the last decade has expanded toward using genetically encoded optical indicators to examine hair-cell function in zebrafish (Kindt et al., 2012; Esterberg et al., 2014; Zhang et al., 2016, 2018; Sheets et al., 2017). These studies have utilized transgenic zebrafish that express functional indicators in either hair cells or afferent neurons using cell-type specific promoters (**Figures 3E–G**). An important advantage of functional imaging over electrophysiogical recordings is the ability to resolve activity spatially among multiple cells and subcellularly within individual cells. In contrast, microphonics or extracellular afferent recordings are the readout of many hair cells within a neuromast, and whole-cell recordings examine activity from one hair cell at a time.

The majority of functional imaging studies have used genetically encoded indicators of calcium because calcium influx is an important feature of both apical mechanotransduction and basal neurotransmission in hair cells (**Figure 1C**). Initial work primarily used genetically encoded calcium indicators localized to the cytosol of lateral-line hair cells. During fluid-jet stimulation, it was shown that calcium signals could be reliably measured in the cytosol (Kindt et al., 2012; Sheets et al., 2012, 2017; Sebe et al., 2017). Disrupting hair-cell mechanotransduction abolished these cytosolic signals indicating that they are mechanically evoked calcium signals. In addition, it was possible to determine the contribution of presynaptic calcium channels to these cytosolic calcium responses by using pharmacology or mutant analysis (Kindt et al., 2012; Sheets et al., 2012, see the genetics section below).

Unfortunately, it is challenging to use a cytosolic calcium indicator to understand the physiological properties of channel activity within hair cells. Therefore, more recent studies have used localized calcium indicators to examine subcellular activity within hair cells (Sheets et al., 2017; Zhang et al., 2018). In particular, membrane-localized calcium indicators have proved advantageous to assess localized calcium influx into the hair-cell bundle and at the hair-cell synapse (Zhang et al., 2018). This approach is particularly useful because both of these measurements can be made within the same cell by using either an apical or basal imaging plane respectively (**Figures 3E,E**’). To determine the location of synaptic ribbons and measure presynaptic calcium signals at sites of release, calcium indicator lines can be combined with an additional transgenic line that marks synaptic ribbons via a Ribeye-mCherry fusion protein (*Tg[myo6b:ribeye-mcherry];* **Figures 3E–G**; Sheets, 2017; Zhang et al., 2018). Similar to work in hair cells, membrane-localized calcium indicators have also been used to assay postsynaptic activity in the afferent process beneath neuromast hair cells (**Figure 3G**). In response to fluid-jet stimulus, afferent calcium signals can be detected at sites adjacent to synaptic ribbons that are marked using a Ribeye-mCherry transgenic line (Zhang et al., 2018).

While calcium indicators have proved useful, other indicators of activity have been utilized to examine hair-cell synapse function in zebrafish. For example, a genetically encoded indictor called SypHy has been used as a readout of vesicle fusion at synaptic ribbons (**Figure 3F**; Zhang et al., 2018). In the future, SypHy may provide valuable information regarding the spatial properties of vesicle fusion at hair-cell synapses. At the postsynapse, instead of a calcium indicator, recent work has used a genetically encoded glutamate sensor to measure postsynaptic activity (Pichler and Lagnado, 2018). Overall, these functional studies highlight that the zebrafish is an excellent model to test the efficacy of using genetically encoded optical indicators, with the hope that they can ultimately be used in both zebrafish and mammalian models to assess hair-cell synapse function. With the advance of functional imaging, it also will be informative to combine electrophysiology and functional imaging in zebrafish to gain a more comprehensive understanding of the temporal and spatial properties underlying hair-cell synapse function.

## The Genetics of Hearing Loss Affecting Hair-Cell Synapses in Mammals and Zebrafish

To date, numerous genetic studies in zebrafish, mice and humans have uncovered molecules required for hearing and balance (Nicolson, 2005; Safieddine et al., 2012; Pan and Holt, 2015). In zebrafish, mutants with hearing and balance defects were first identified behaviorally in large-scale forward genetic screens (Nicolson et al., 1998; Lin et al., 2016). These fish were initially identified as motility mutants with circling behavior, an indicator of vestibular dysfunction; additional screening showed these fish also lacked acoustic-vibrational startle responses, an indicator of deafness (Nicolson et al., 1998; Tubingen 2000 Screening Consortium). Over the last 20 years considerable work has focused on identifying the lesions underlying deafness in these zebrafish mutants. Concurrent work in mice and humans has revealed, perhaps not surprisingly, that orthologous genes, when mutated, cause deafness in zebrafish and mammals. This body of work supports functional conservation of deafness genes among vertebrates.

During characterization of these genes, zebrafish deafness mutants were classified based on morphological and functional assays. For example, one class of mutations disrupted overall hair-cell morphology while another class specifically affected hair-bundle integrity. In most zebrafish mutants with disrupted hair-bundle integrity, mechanotransduction was also affected (Ernest et al., 2000; Seiler et al., 2004; Söllner et al., 2004; Gleason et al., 2009). Importantly, a distinct class of zebrafish mutants had normal hair-cell and hair-bundle morphology and intact mechanotransduction, indicating that that the affected genes altered function downstream of hair-bundle function. Further characterization of 6 of these zebrafish mutants revealed molecules that are specifically required for proper hair-cell neurotransmission: Ca_V_1.3, Vglut3, Nsf, Rabconnectin 3α, Synaptojanin and Wrb (Sidi et al., 2004; Obholzer et al., 2008; Trapani et al., 2009; Mo and Nicolson, 2011; Einhorn et al., 2012; Lin et al., 2016).

In the section below, we outline how studies characterizing zebrafish auditory and vestibular mutants revealed the function of these molecules in hair-cell neurotransmission. Further, we discuss how these mutants have expanded our understanding of each molecule’s contribution to hair-cell synaptic development, maintenance and function. To date, mutations in four of the synapse-associated genes identified above have also been associated with human hearing loss (Rodríguez-Ballesteros et al., 2008; Ruel et al., 2008; Baig et al., 2011). This genetic conservation between zebrafish and humans suggests that there is also functional conservation at hair-cell synapses, and further supports that the zebrafish model is useful for studying auditory synaptopathy.

### *Ca*_V_*1.3;* Sinoatrial Node Dysfunction and Deafness (SANDD) Syndrome

In humans, loss of function mutations in Ca_V_1.3 (CACNA1D) results in Sinoatrial Node Dysfunction and Deafness (SANDD), a disorder whereby affected individuals have abnormal heart rhythms and severe deafness (Platzer et al., 2000; Baig et al., 2011). Ca_V_1.3 channels are the presynaptic calcium channel required for neurotransmission at hair-cell synapses. The requirement for Ca_V_1.3 channels in hearing is highly conserved; mutations in *ca_V_1.3* result in profound deafness in human, mice and zebrafish (Sidi et al., 2004; Brandt et al., 2005; Baig et al., 2011).

Ca_V_1.3 channels are part of the L-type calcium channel family—they are uniquely sensitive to dihydropyridines and have large single channel conductance (Bean, 1989). Ca_V_1.3 channels also activate with rapid kinetics at low voltages relative to other Ca_V_1 channels (Lipscombe et al., 2004) and inactivate slowly in hair cells. These properties make Ca_V_1.3 channels ideal for mediating rapid and continuous exocytosis. In zebrafish and mice, Ca_V_1.3 channels cluster tightly at synaptic ribbons (Frank et al., 2009, 2010; Sheets et al., 2011; Wong et al., 2014). Presynaptic clustering of Ca_V_1.3 channels is thought to be important to tightly couple calcium influx and vesicle release. Precise control of vesicle release is an important feature for reliable sensory encoding in hair cells (Brandt et al., 2003, 2005; Wong et al., 2014).

In addition to its role in hair-cell neurotransmission, Ca_V_1.3 channels are essential for hair-cell development and synapse maintenance. While *ca_V_1.3* knockout mice initially form synapses, they progressively lose hair cells and postsynaptic afferents that innervate the remaining hair cells degenerate (Glueckert et al., 2003). In zebrafish *ca_V_1.3a* mutants, lateral-line hair cells were also shown to initially form synapses, a phenotype that can be assessed in developing hair cells when larvae are 3-days-old, 2 days prior to the onset of lateral-line function (Trapani and Nicolson, 2010; Suli et al., 2016). Nevertheless, in the zebrafish, a progressive loss of juxtaposition between hair-cell pre- and postsynaptic components was observed in functionally mature hair cells of *ca_V_1.3a* mutants just 2 days later (Sheets et al., 2012). Additionally, this work found hair-cell ribbons were significantly enlarged in both 3- and 5-day-old *ca_V_1.3a* mutants. The rapid formation (by day 3) and subsequent loss of synaptic juxtaposition (at day 5) in zebrafish *ca_V_1.3a* mutants is one example of how quickly phenotypic differences in synapse development and maintenance can be assayed in zebrafish. By taking advantage of these distinct developmental time points, this study also found that transiently (1 h.) treating the developing hair cells of 3 day-old zebrafish with dihydropyridine agonists and antagonists was able to rapidly decrease or increase presynaptic-ribbon size respectively (Sheets et al., 2012). In 5 day-old zebrafish, these compounds affected synaptic-ribbon size to a far lesser degree, revealing that calcium influx through Ca_V_1.3 channels could dramatically influence presynaptic morphology during a critical window of development.

Cumulatively, these results support that functional Ca_V_1.3 channels are necessary to properly form synaptic ribbons and to maintain pre- and postsynaptic juxtaposition in zebrafish hair cells. Although is it clear that Ca_V_1.3-dependent presynaptic calcium influx regulates presynaptic size during zebrafish hair-cell development, it is less clear what role these channels play in synapse maintenance. It has been proposed that synapse maintenance could require release of synaptic vesicle contents (Mo and Nicolson, 2011). Consistent with this idea, zebrafish *ca_V_1.3a* mutants lack evoked and spontaneous synaptic vesicle release (Trapani and Nicolson, 2011). Because formation and maintenance of hair-cell synapses in mutants lacking synaptic glutamate release appear relatively normal (*vglut3*-/-; see next section), it possible that the release of trophic factors from the synapse is required for maintenance of hair-cell synaptic connections (Fritzsch et al., 1997, 2004; Mo and Nicolson, 2011; Kersigo and Fritzsch, 2015). In the future, it may be informative to use zebrafish as a platform to screen for compounds that maintain synaptic juxtaposition in *ca_V_1.3a* mutants in order to identify factors necessary for hair-cell synapse maintenance.

### *SLC17A8/vglut3;* Autosomal Dominant Deafness-25 (DFNA25)

In human patients, autosomal dominant deafness-25 (DFNA25) is a progressive, high frequency non-syndromic hearing loss caused by a heterozygous mutation in the *SLC17A8* gene encoding Vesicular Glutamate Transporter-3 (VGLUT3) (Ruel et al., 2008). Vglut3—a transporter that packages glutamate into synaptic vesicles—was identified independently in both zebrafish loss-of-function mutants and mouse knockouts as a critical component for hearing and hair-cell neurotransmission (Obholzer et al., 2008; Seal et al., 2008). These studies found that both zebrafish hair cells and mammalian auditory hair cells express *vglut3*. In zebrafish and mice, *vglut3* mutant hair cells have normal microphonic potentials, suggesting that Vglut3 is not required for mechanotransduction (Obholzer et al., 2008; Ruel et al., 2008). Additionally, *vglut3* mutant mice and zebrafish show normal calcium responses, and in mice exocytosis is not altered. In zebrafish, despite normal mechanotransduction and evoked calcium responses in hair cells, no postsynaptic spikes are detected in the innervating afferent neurons (Obholzer et al., 2008; Sheets et al., 2012). Similarly, in *vglut3* knockout mice, auditory nerves lacked responses to auditory stimuli, despite normal hair-cell calcium currents and exocytosis (Ruel et al., 2008).

In *vglut3* zebrafish mutants, afferent innervation appears relatively normal (Sheets et al., 2012). There does not appear to be any major structural changes in presynaptic ribbon morphology beyond the normal variances in mice and zebrafish (Obholzer et al., 2008; Ruel et al., 2008). Overall, these mild synaptic morphology phenotypes in *vglut3* mutants are quite different compared to *ca_V_1.3* mutants where the synapses ultimately degenerate. Synapses may be preserved due to the presence of normal calcium currents and hair-cell exocytosis despite an absence of glutamate release (Ruel et al., 2008). This supports an important role for exocytosis in the release of other trophic factors in synaptic maintenance. Relatively normal development and maintenance of hair cells in *vglut3* knockout mice have made this deafness model a promising target of virally-mediated gene therapy (Akil et al., 2012).

Notably, there are a few morphological differences observed between Vglut3-deficient zebrafish lateral-line hair cells and Vglut3-deficient mouse auditory hair cells. Zebrafish *vglut3* mutant synaptic ribbons have a reduced number of ribbon-associated vesicles, which is not observed in mouse auditory hair cells. Additionally, there is evidence that the glutamate transporter Vglut1 is also expressed in zebrafish hair cells, yet Vglut1 appears unable to compensate for neurotransmission in the *vglut3* mutant (Obholzer et al., 2008). Further studies in zebrafish are needed to confirm the presence of Vglut1 in hair cells and to define its function in order to understand the unique functional role of Vglut3 in hair-cell neurotransmission.

### *DMXL2/Rabconnectin 3α;* Autosomal Dominant Deafness-71 (DFNA71)

A recent study identified a heterozygous missense variant of *DMXL2* that is associated with dominant, non-syndromic hearing loss in humans (Chen et al., 2017). Notably, this gene had been previously identified in a zebrafish hearing and balance mutant and represents an example of hereditary deafness gene identified in zebrafish prior to mice or humans. *DMXL2* encodes Rabconnectin 3α—the α-subunit of the Rabconnectin protein complex. *Rbc3α* zebrafish mutants, when compared to *vglut3* and *ca_V_1.3a* zebrafish mutants, have relatively mild to moderate auditory and vestibular deficits (Einhorn et al., 2012). Phenotypically, similar to *vglut3* mutants, both pre- and post-synaptic morphology appeared normal in *rbc3α* mutants. Subsequent analysis of Rbc3α localization revealed it was enriched basolaterally and overlapped with Vglut3 in hair cells, suggesting that Rbc3α is localized to synaptic vesicles.

This study found that zebrafish hair cells deficient in Rbc3α impacted Vacuolar-type H^+^-ATPase (V-ATPase) localization at the base of hair cells (Einhorn et al., 2012). V-ATPase generates a proton gradient and acidifies subcellular compartments, including synaptic vesicles. This gradient is important for the accumulation of glutamate into synaptic vesicles. These results suggested that *rbc3α* mutants could have deficient synaptic-vesicle acidification. To determine whether vesicles properly acidified, this study pioneered the use of lysotracker in hair cells. Lysotracker is a membrane-permeable vital dye that labels acidic organelles. In combination with a live presynaptic ribbon label (Ribeye-GFP), lysotracker brightly labeled rings around synaptic ribbons that likely correspond to ribbon-associated vesicles. The intensity of lysotracker labeling was dramatically reduced in *rbc3α* mutants, indicating reduced acidification of organelles and vesicles surrounding synaptic ribbons.

To better understand the relationship between Rbc3α and the V-ATPase, this work used the genetic tractability of the zebrafish model to rapidly express tagged proteins in hair cells. Transient expression of either the cytosolic (V1) or the membrane (V0) subunits of the V-ATPase in wild-type and Rbc3α deficient hair cells revealed that Rbc3α is required to traffic or assemble the V1 subunit at the base of hair cells (Einhorn et al., 2012). Therefore, Rbc3α is required for proper V-ATPase localization and ultimately synaptic vesicle acidification. Because synaptic vesicle acidification contributes to vesicular glutamate accumulation, this observation suggests that *rbc3α* mutant synaptic vesicles contain less glutamate. Accordingly, afferent recordings revealed reduced glutamate-dependent evoked release from *rbc3α* mutant hair cells. In addition, *rbc3α* mutants showed loss of fidelity of phase-locked spiking at higher frequencies (20 vs. 60 Hz) (Einhorn et al., 2012). Currently there is no other established model to study *DMXL2*-related human hearing loss. In the future, it will be interesting to test whether expression of the dominant human mutation in zebrafish hair cells also disrupts hearing and balance, which could provide further insight into the pathology underlying this genetic lesion.

### Synaptojanin

Proteins in the Synaptojanin family are lipid phosphatases that play an important role in endocytosis and vesicle recovery at synapses (Harris et al., 2000; Song and Zinsmaier, 2003). Although no members of the Synaptojanin family have been associated with hearing loss in humans, mutations that abolish the lipid phosphatase activity of Synaptojanin 2 result in progressive age-related hearing loss in mice without any other accompanying phenotype (Manji et al., 2011). Synaptojanin 2 is expressed in auditory hair cells, and the progressive hearing loss observed in *Synj2* mutants appears to be due to degeneration or loss of hair bundles, and sunken appearance of cell bodies, followed by hair-cell death. These observations support that Synaptojanin 2 plays an important role in hair-cell survival, but the process by which it contributes to cell survival is not understood.

While mice express Synaptojanin 2 in hair cells, zebrafish express Synaptojanin 1 (McDermott et al., 2007; Trapani et al., 2009). *Synj1* zebrafish mutants, similar to *rbc3α* zebrafish mutants, have moderate auditory and vestibular defects. These behavioral defects were accompanied by morphological disruptions in Synj1-defficient hair cells; specifically, basal membrane protrusions, or blebbing. These protrusions were dependent on functional Cav1.3 channels and were observed in 1/3 of *synj1* mutant hair cells. In contrast to mice *Synj2* mutants, other aspects of hair-cell morphology in zebrafish *synj1* mutants appeared normal. Transmitted electron micrographs revealed fewer vesicles at *synj1* ribbons, indicating vesicle recycling was impaired. Reduced vesicles and basal membrane protrusions in mutant *synj1* hair cells led to deficits in synapse function; afferent recordings in *synj1* mutants revealed a delay in afferent spike timing and impaired phase-locking in response to high-frequency stimuli (Trapani et al., 2009). Speculatively, impaired hair-cell membrane recycling could contribute to a progressive degeneration of hair cells. It will be informative in follow-up studies to examine whether disrupting Synj1 function impacts zebrafish hair-cell maintenance and survival in older larvae.

### *Otoferlin and *WRB (pwi)*;* Autosomal Recessive Deafness-9 (DFNB9)

In human patients, mutations in the Otoferlin gene gives rise to neurosensory non-syndromic recessive deafness DFNB9 (Yasunaga et al., 1999), and is the cause of ∼1.4–5% of the cases of autosomal recessive hearing loss (Santarelli et al., 2015). Otoferlin exists in a long form containing six C2 domains (C2A-F) and a short form containing three C2-domains (Yasunaga et al., 2000). C2 domains are important for membrane localization and bind calcium (Lek et al., 2012). Mutations in nearly any of the C2 domains in the long form (C2B, C, D, E, or F) are linked to deafness in humans and mice (Yasunaga et al., 2000; Ramakrishnan et al., 2009), indicating that the presence of the long form is important for hearing. Knockdown of Otoferlin in zebrafish results in deafness (Chatterjee et al., 2015). Otoferlin is proposed to be an essential regulator of hair-cell neurotransmission, functioning to both couple calcium signaling with vesicle fusion and to regulate vesicle replenishment (Roux et al., 2006; Pangršič et al., 2015; Vogl et al., 2016; Michalski et al., 2017). Otoferlin’s sequence identity and protein localization are highly conserved between divergent species (Goodyear et al., 2010). Additionally, Otoferlin’s function also appears to be conserved; acoustic startle responses can restored in zebrafish *otof* knockdowns using exogenous mouse Otoferlin (Chatterjee et al., 2015).

A highly conserved role for Otoferlin in hair-cell synapse function is further supported by the identification of a gene important for hearing and vision in a large-scale mutagenesis screen. A null mutation the gene *pinball wizard* (*pwi*) resulted in zebrafish with impaired acoustic startle response, vestibular abnormalities and defective optokinetic response (Lin et al., 2016). *Pwi* encodes tryptophan-rich basic (WRB) protein, a small transmembrane protein found in the endoplasmic reticulum that is a receptor for insertion of tail-anchored (TA) proteins. Disruption of TA-protein membrane insertion would likely result in disruption of TA-protein trafficking, and numerous hair-cell vesicular proteins are TA, including Otoferlin. A subsequent report further examined zebrafish *pwi* mutants, and verified that Wrb is necessary for normal Otoferlin protein levels in hair cells and hearing in zebrafish (Vogl et al., 2016). Additionally, this study showed mutating *wrb* in mice disrupted ER-insertion of Otoferlin into vesicles, which greatly reduced Otoferlin levels in auditory hair cells. A reduction in Otoferlin levels contributed to impaired sustained exocytosis at *Wrb*-deficient hair-cell synapses and disruptions in sound encoding. These observations further support the functional conservation of hair-cell synaptic proteins between zebrafish and mammals. This works also demonstrates the effectiveness of using the zebrafish model to identify novel proteins involved in auditory synaptopathy and to define the molecular functions of these proteins in hair cells.

### Ribeye

Ribeye is the main component of synaptic ribbons and is a presynaptic protein that is unique to ribbon synapses (Schmitz et al., 2000). Ribeye is a splice isoform of the transcriptional co-repressor CtBP2. As CtBP2 is a protein that regulates a number of diverse transcriptional targets, knockouts of CtBP2 are embryonic lethal (Hildebrand and Soriano, 2002). Biochemical studies have demonstrated that individual Ribeye subunits self-associate, and this self-association may form synaptic ribbons (Magupalli et al., 2008). There is currently no known mutation in CtBP2/Ribeye that contributes to hearing loss in humans. Nevertheless, studies in zebrafish and mouse models have depleted or knocked out Ribeye expression (while leaving the transcriptional co-repressor CtBP2’s function intact) to understand the role of the synaptic ribbon in hair-cells. These genetic studies motivated discussion of Ribeye in this section.

In zebrafish, there are two paralogs of Ribeye and both are found in hair cells. Two main studies have examined the role of these Ribeye paralogs in zebrafish hair cells; one study transiently knocked down Ribeye and the other study created a genetic mutant that permanently eliminated nearly all Ribeye in hair cells. Transient knockdown of both Ribeye transcripts during development resulted in reduced number of hair-cell synaptic ribbons which correlated with reduced afferent innervation and reduced afferent firing (Sheets et al., 2011). By contrast, while *ribeye* mutant zebrafish also eliminated synaptic ribbons, these genetic mutants did not appear to affect afferent innervation nor significantly disrupt lateral-line afferent firing properties (Lv et al., 2016). This latter work suggests that compensatory mechanisms may be engaged when Ribeye is severely and permanently depleted. One additional similarity observed with either transient Ribeye knockdown and *ribeye* mutants was that Ca_V_1.3a channels failed to localize and cluster at the synapse. Despite this clustering defect, an enhancement of Ca_V_1.3a channel currents was observed in *ribeye* mutants (Lv et al., 2016).

In the mouse knockout of Ribeye, the entire ribbon structure was shown to be absent in hair cells (Maxeiner et al., 2016; Becker et al., 2018; Jean et al., 2018). Yet the absence of synaptic ribbons in knockout mice did not disrupt Ca_V_1.3 localization at the hair-cell synapse. Instead, Ribeye was shown to be important for presynaptic Ca_V_1.3 calcium channel organization; without Ribeye there was a preponderance of small Ca_V_1.3 clusters at each synapse instead of a single organized structure (Jean et al., 2018). Functionally, Ribeye knockout mice showed minor auditory deficits despite the absence of synaptic ribbons in hair cells (Becker et al., 2018; Jean et al., 2018). Both zebrafish and mouse studies cumulatively revealed that loss of synaptic ribbons via mutation or knockout of Ribeye leads to surprisingly minor deficits hair-cell synapse function and support the idea that compensatory mechanisms exist in both model systems.

In addition to these loss of function models, work in zebrafish has also demonstrated that overexpression of exogenous Ribeye in hair cells can enlarge synaptic ribbons and influence synaptic activity (Sheets et al., 2017). Synaptic ribbons in hair cells overexpressing Ribeye were ∼2 fold larger and transmission electron micrographs showed that these synaptic ribbons had a greater number of synaptic vesicles relative to wild-type siblings. Hair cells containing enlarged synaptic ribbons had less tightly clustered Ca_V_1.3a channels yet showed increased Ca_V_1.3a channel currents and correspondingly larger ribbon-localized calcium signals. Despite larger calcium signals, there was no change in exocytosis or afferent spike number in response to strong stimulus. Importantly, enlarged synaptic ribbons resulted in a significant reduction in spontaneous afferent activity, and disrupted evoked release at the onset of stimuli. These results indicate that enlarging the synaptic ribbon can influence the activity of innervating afferent neurons and degrade sensory encoding. These observations may have clinical significance; in noise-exposed guinea pig, the synaptic-ribbon size gradient found in auditory hair cells is disrupted (Furman et al., 2013) and synaptic ribbon volume is increased (See section on noise-exposure below, Furman et al., 2013; Song et al., 2016). This increase in synaptic ribbon volume is accompanied by deficits in intensity and temporal coding by auditory nerve fibers (Song et al., 2016). An interesting prospect for future zebrafish work is to determine whether noise can also induce changes in synaptic ribbon size and whether these changes can influence afferent neuron function, and if so by what mechanisms.

## The Future of Using Zebrafish Genetics to Study Hair-Cell Synapses

Action potentials do not drive neurotransmitter release at hair-cell synapses. Instead, in order to convey information about timing and intensity of stimuli, hair-cell neurotransmission is driven by graded depolarizations (Glowatzki and Fuchs, 2002; Trussell, 2002). While hair-cell synapses contain many of the same molecular components as conventional synapses, such as presynaptic calcium channels and postsynaptic glutamate receptors, their specialized function may require synaptic proteins that are unique to hair cells. Indeed, a number of molecules that are required at neuronal synapses are not present in mammalian hair cells, including Munc-13 and CAPS (two important proteins for synaptic vesicle tethering and priming), Synaptotagmins 1 and 2 (calcium sensors for vesicle fusion), Complexins (which regulate vesicle fusion), and Synaptophysins (Safieddine and Wenthold, 1999; Strenzke et al., 2009; Johnson et al., 2010; Uthaiah and Hudspeth, 2010; Vogl et al., 2015). These important synaptic functions are instead thought to be accomplished by specialized hair-cell synaptic proteins. One notable example is Otoferlin which, as highlighted in this review, appears to act in place of Synaptotagmins and functions as a calcium sensor for vesicle fusion (Johnson and Chapman, 2010; Michalski et al., 2017).

Identifying the unique molecular players at ribbon synapses in hair cells has been hampered by the difficultly in acquiring a sufficient amount of material for biochemical and proteomic approaches (Uthaiah and Hudspeth, 2010; Kantardzhieva et al., 2012). Moreover, it is possible that proteins found at both conventional and hair-cell synapses may be present but not be functioning in the same way. For example, SNARE proteins that are required for vesicle fusion at conventional synapses may also be present in hair cells (Uthaiah and Hudspeth, 2010), but do not appear to be required in mouse hair cells for synaptic-vesicle fusion (Nouvian et al., 2011). In future studies, with the advent of CRISPR technology, zebrafish could be used as platform to rapidly and inexpensively identify what synaptic proteins are present in hair cells. Using this approach, it may be possible to identify the molecular equivalents of neuronal molecules that are not present in hair cells, and determine whether neuronal synaptic proteins have specialized functions when they are present in hair cells. In support of this idea, a recent study in zebrafish demonstrated that targeted mutagenesis of protein-coding genes using CRISPR-Cas9 is a powerful and high-throughput way to assess the role of candidate deafness genes identified in humans (Varshney et al., 2015). This work also highlights that the zebrafish model is a useful platform to not only rapidly evaluate the role of both known human deafness genes but also probe for yet unknown molecules that may be required at hair-cell synapses. CRISPR technology, combined with the functional and morphological toolkit outlined in this review, make zebrafish a favorable model to use toward determining the complete molecular composition of hair-cell synapses.

## Noise Exposure, Excitoxicity and Acquired Hearing Loss in Zebrafish

In addition to gene mutations that cause hereditary forms of hearing loss, environmental factors such as intense or prolonged noise exposure can result in an acquired form of hearing loss. In humans, intense noise exposure can rapidly lead to profound hearing loss. In other cases following noise exposure, hearing loss is not profound but rather hearing sensitivity is diminished and higher sound pressure levels are needed to perceive a given stimulus (Mills et al., 1979). This diminished hearing sensitivity for a given stimulus is referred to as an elevated shift in hearing threshold.

After noise exposure, hearing loss can either be permanent or temporary depending on the intensity, duration and repetition of the exposure. Cumulatively, studies in mammals have demonstrated that noise exposures can result in damage or loss of hair cells, hair-cell synapses or the innervating afferent neurons (Bohne, 1976; Dinh et al., 2015; Liberman and Kujawa, 2017). Intense noise exposures that result in permanent hearing loss are accompanied by progressive hair-cell death and loss of afferent neurons (Ryan et al., 2016). Notably in mice, intense impulse noise (i.e., blast) also appeared to result in a significant decrease in the number of hair-cell synapses in surviving hair cells (Cho et al., 2013). This synaptic pathology supports the hypothesis that intense noise exposures contributes to both hair-cell damage, synapse loss and ultimately a permanent, acquired hearing loss.

By contrast, moderate noise exposures are initially accompanied by elevated shifts in hearing threshold, but the thresholds eventually return to normal (Ryan et al., 2016). These noise exposures leave auditory hair cells intact, but contribute to afferent terminal swelling and a subsequent reduction in synaptic contacts, followed by progressive loss of auditory nerves (Kujawa and Liberman, 2009; Lin et al., 2011; Jensen et al., 2015). Currently it is hypothesized that, although clinical hearing thresholds return to normal, there may be subclinical hearing deficits associated with hair-cell synapse loss and afferent nerve degeneration (Bharadwaj et al., 2014). These deficits include difficultly resolving sounds in challenging listening environments such as discerning speech in a noisy room (Moser and Starr, 2016). Afferent-terminal swelling and synapse loss are thought to be a consequence of excess glutamate accumulation in the synaptic cleft during noise over-exposure, resulting in glutamate excitotoxicity (Puel et al., 1994, 1998; Hakuba et al., 2000; Ruel et al., 2005). Similar to genetic lesions that impair hair-cell synapse function, acquired noise-induced hearing loss resulting from a reduction in hair-cell synapses is a form of auditory synaptopathy (Moser and Starr, 2016).

### Intense Noise Exposure in Zebrafish

While zebrafish have been used extensively to understand how ototoxic agents, such as aminoglycoside antibiotics and platinum-based cancer therapeutics, damage hair-cell organs (Coffin et al., 2010; Namdaran et al., 2012; Ou et al., 2012), less work has been done to model the toxic effects of noise damage on these organs. Currently there are only a few published studies exploring noise exposure paradigms in zebrafish (Schuck and Smith, 2009; Sun et al., 2011; Uribe et al., 2018). In studies examining auditory over-stimulation, adult zebrafish were exposed to a 100 Hz pure tone at 179 dB for 36 h. After this intense exposure, hair cells in the saccular epithelia (a hair-cell organ in the zebrafish inner ear used to detect sound (Schuck and Smith, 2009)) showed damage or loss of apical mechanosensory structures immediately following noise exposure. The damage was most apparent in the caudal region of the saccule which corresponds to a region sensitive to low frequency tones. Overall, the cellular damage following intense noise exposure was similar to damage that has been observed in mammalian models (Wang et al., 2002). Similar to the regenerative capability that has been demonstrated in the lateral-line system (Coffin et al., 2010; Namdaran et al., 2012; Ou et al., 2012), there was evidence of hair-cell proliferation in the adult zebrafish inner ear just days after noise exposure. Follow up work from this study later revealed that Growth Hormone (GH) may be important for hair-cell proliferation after this level of trauma (Schuck and Smith, 2009; Sun et al., 2011).

The role of GH is intriguing because work in mammals indicates that other hormones and neurotrophins including Thyroid hormone (TH), Neurotrophin-3 (NT-3), Brain-derived neurotrophic factor (BDNF) may be important for the survival and recovery of afferent terminals and synapses (Wan and Corfas, 2015). More specifically, studies in noise-exposed mice suggest NT-3/TrkC signaling promotes synaptic repair and regeneration in auditory hair cells (Wan et al., 2014; Suzuki et al., 2016), while BDNF/TrkB signaling regulates time-dependent noise sensitivity and protects against synapse loss during periods of wakefulness (Meltser et al., 2014). In zebrafish, it has been demonstrated that hair-cell synapse stabilization during normal development requires the protein *N*-ethylmaleimide-sensitive factor (Nsf) in order to release neurotrophic factors including BDNF (Mo and Nicolson, 2011). Future studies in the zebrafish model could provide mechanistic information toward how these trophic factors provide protection or promote repair of hair-cell synapses following noise exposure.

### Modeling Noise-Induced Excitotoxicity in Zebrafish

In addition to the few noise exposure studies in zebrafish, recent work has used pharmacology to model glutamate excitotoxicity associated with noise exposure (Sebe et al., 2017; Sheets, 2017). These models are based on the premise that, during noise exposure, excess glutamate accumulates in the synaptic cleft leading to over activation of ionotropic glutamate receptors (iGluR) and subsequent excitotoxic damage. Application of the iGluR agonists α-amino-3-hydroxy-5-methyl-4-isoxazolepropionic acid (AMPA), Kainic acid (KA) and *N*-methyl-D-aspartate (NMDA) to mammalian inner ears or hair-cell explants has also been used to mimic glutamate excitotoxicity associated with noise exposure. In mammals, application of AMPA or KA results in overactivation of the iGluR receptors mediating neurotransmission on postsynaptic afferent terminals (Puel et al., 1994; Zheng et al., 1997; Sun et al., 2001; Sasaki et al., 2012). Subsequently, the afferent terminals swell and, in the case of KA application on auditory hair-cell explants, neurites retract (Wang and Green, 2011). In cases of AMPA administration to inner ears, a small but significant percentage of inner hair cells are also lost 7 days following exposure (Hakuba et al., 2003; Hyodo et al., 2009). Two recent pharmacological studies have used iGluR agonists to mimic noise exposure in zebrafish larvae. These studies took advantage of the accessibility of the larval lateral-line organ to apply iGluR agonists externally, and image hair cells during and after drug exposure (Sebe et al., 2017; Sheets, 2017).

In one study, a single, short application of AMPA (100 or 300 μM AMPA, 15 min) was examined (Sebe et al., 2017). This treatment resulted in swelling of lateral-line afferent nerve terminals. This swelling was similar to what has been reported in mammal auditory system after AMPA exposure (Puel et al., 1994; Hyodo et al., 2009). Functional calcium imaging revealed a loss of activity in afferent nerve terminals after this treatment. Lateral-line hair cells, on the other hand, were not morphologically affected by these treatments and showed normal mechanotransduction, suggesting glutamate excitotoxicity underlies afferent terminal damage and synaptopathy (Sebe et al., 2017). Importantly, this work demonstrated that the excitotoxic effects of AMPA occurred through calcium-permeable AMPA receptors (CP-AMPARs), as blocking CP-AMPARs prevented postsynaptic swelling and loss-of-function. Furthermore, this study used immunohistochemistry, electrophysiology and pharmacology to demonstrate that CP-AMPARs are present and mediate synaptic currents not only within the postsynapses of lateral-line afferents of the zebrafish lateral line, but also within rat and bullfrog auditory synapses (Sebe et al., 2017). The morphological and functional conservation of CP-AMPARs among species indicates the mechanism underlying glutamate excitotoxicity at hair-cell synapses may also be conserved.

A second study used similar methodology but applied iGluR agonists for longer durations (Sheets, 2017). Here the iGluR agonist NMDA or non-sensitizing AMPA/kainite receptor agonist KA were applied over longer time scales (10–600 μm; 1 h). Similar to what has been demonstrated in the mammalian auditory system after NMDA and KA application (Puel et al., 1994), this study found that NMDA did not cause appreciable swelling in the afferent nerve terminals, while KA was extremely potent and caused swelling and even bursting of afferent nerve terminals. In addition, exposure to either NMDA or KA induced apoptotic hair-cell death in a dose-dependent manner (Sheets, 2017). Remarkably, hair-cell death was independent of damage to post-synaptic terminals—loss of hair cells following NMDA and KA application occurred even in the absence of afferent neurons. Further, this work identified AMPA, Kainate and NMDA receptor subunits that appear to be expressed in hair cells, suggesting that presynaptic iGluR receptors may contribute to hair-cell excitotoxic damage.

## The Future of Noise Exposure and Excitotoxicity Research in Zebrafish

Currently there are no published zebrafish studies in adults or larvae using noise exposures to model acquired auditory synaptopathy in the ear. In the future, it will be useful to create protocols to modulate the intensity and duration of noise exposures in order to define the pathological changes in the zebrafish inner ear that are associated with moderate noise exposure. By modulating the intensity and duration of noise exposures in zebrafish, it will be possible to examine the dynamic progression of damage following moderate noise exposure, including loss of afferent fibers and synapses (Kujawa and Liberman, 2009; Shi et al., 2013; Song et al., 2016). In addition to noise damage paradigms to study pathology in the zebrafish inner ear, it will be experimentally worthwhile to develop approaches to directly mechanically over-stimulate the well characterized lateral-line organs. Here, noxious water flow could be used to over-stimulate lateral-line hair cells. Although a previous report outlined a microfluidic device that could be used to confer damage to the lateral-line system in larval zebrafish, no studies have demonstrated the effectiveness of this design (Kwon et al., 2014).

After establishing both moderate and intense noise exposure methods in zebrafish, it will be informative to apply the same tools and assays that have been used to understand the effects ototoxic drugs on hair-cell pathology. For example, the zebrafish lateral line has been used to screen for compounds that protect hair cells or promote hair-cell regeneration during and after ototoxic insult (Coffin et al., 2010; Ou et al., 2012; Esterberg et al., 2014, 2016; Hailey et al., 2017). Based on ototoxicity studies, it is likely that the zebrafish lateral-line system or inner ear could also be used as a screening platform to identify compounds that are protective during noise exposure or lateral-line over-stimulation. Alternatively, zebrafish could be used to identify compounds that promote synaptogenesis after synapse loss and afferent nerve damage.

These studies will be particularly advantageous in larval zebrafish where transgenic lines (**Figure 3**), can be used to image hair cells *in vivo*. Using live imaging, it will be possible to examine the morphology of hair cells, ribbon synapses and afferent nerve terminals during noise exposure, as well as during repair and regeneration. Currently, it is not possible to visualize these changes in the hair-cell organs in living mammals. The ability to visualize morphological changes during and after insults in whole animals is an important advantage to using zebrafish for these studies. These live imaging approaches could reveal the specific pathological changes accompanying both moderate and intense noise exposures. While imaging morphological changes accompanying noise exposure or lateral-line over stimulation will be invaluable, it will also be interesting to explore the functional consequences to the synapse during the noxious insult, as well as during recovery. These studies could be accomplished using electrophysiology and imaging-based methods that have been established in the zebrafish lateral line (**Figure 3**).

Finally, in addition to examining the morphological and functional consequences of noise exposure or lateral-line over stimulation in zebrafish, it will also be worthwhile to explore the downstream molecular mechanisms underlying the observed pathologies, as well as the recovery. In recent years, several models and methods have been developed to profile gene expression changes in specific cell types, including zebrafish hair cells (Steiner et al., 2014; Esterberg et al., 2016; Barta et al., 2018; Matern et al., 2018). It will therefore be beneficial and informative to use these approaches to define the pre- and post-synaptic molecular pathways underlying the pathologies during and recovery after noise damage.

## Author Contributions

KK and LS wrote the review article and made the figures.

## Conflict of Interest Statement

The authors declare that the research was conducted in the absence of any commercial or financial relationships that could be construed as a potential conflict of interest.
